# Precision oncology’s translation gap—Can molecular tumor boards bridge it?

**DOI:** 10.1371/journal.pmed.1005165

**Published:** 2026-06-30

**Authors:** Margaret M. Byrne, Jill M. Kolesar

**Affiliations:** 1 College of Medicine, University of Iowa, Iowa City, Iowa, United States of America; 2 College of Pharmacy, University of Iowa, Iowa City, Iowa, United States of America

## Abstract

Molecular tumor boards translate complex genomic data to more effectively inform personalized care, and a new meta-analysis shows they boost clinical outcomes. In this Perspective, Margaret Byrne and Jill Kolesar discuss how we may feasibly and equitably expand the use of MTBs.

## Teaser

Precision oncology is revolutionizing cancer care, but isn’t reaching enough patients. Molecular tumor boards (MTBs) translate complex genomic data to more effectively inform personalized care, and a new meta-analysis shows they boost clinical outcomes. But how can we feasibly and equitably expand the use of MTBs?

Precision medicine has reshaped oncology by enabling treatment selection based on the molecular profile of an individual tumor. This strategy relies on next-generation sequencing (NGS) of tumor or blood samples to identify actionable genomic alterations that may inform targeted therapy. In selected populations, targeted therapies have been associated with improved clinical outcomes, better quality of life, and fewer adverse events compared with conventional treatments [1].

Despite these advances, precision medicine remains underutilized, and lower uptake has been linked to inferior outcomes [2]. Barriers are multifactorial and include reimbursement limitations, variable perceptions of clinical utility, limited clinician training in genomics, and insufficient infrastructure to support interpretation of complex sequencing data [3,4]. Molecular tumor boards (MTBs) have emerged as a possible solution to address these challenges by facilitating the clinical interpretation and application of genomic results.

**Fig 1:**
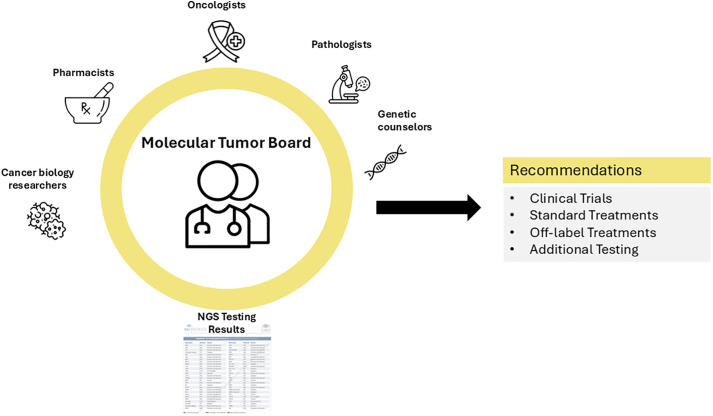
Molecular tumor board composition and workflow. Icons from thenounproject.com (see Acknowledgments).

MTBs are multidisciplinary teams that review NGS results and make treatment recommendations. These MTBs typically include medical oncologists, pathologists, molecular biologists, pharmacists, and genetic counselors (Fig 1). The primary goal of MTBs is to integrate genomic findings with clinical information to generate evidence-informed, patient-specific recommendations, which can include clinical trials, targeted therapies, standard-of-care treatments, or additional diagnostic testing. Effective MTBs share several core features, including multidisciplinary participation, use of standardized frameworks for NGS interpretation, and timely case review to avoid delays in treatment decisions [5].

MTBs are resource-intensive and a central question is whether they meaningfully improve patient outcomes. Until recently, the evidence base was limited, consisting primarily of single-center cohort studies. A systematic review that evaluated 14 studies published through 2020 found evidence that MTBs can improve clinical outcomes, while also highlighting the need for higher-quality data and greater standardization in approaches and outcome measures [6].

Evidence from meta-analyses further suggests that MTBs may improve clinical outcomes, although the strength of prior evidence varies. In a meta-analysis including 34 studies, Gladstone and colleagues showed MTB-recommended treatments improved both overall survival and progression-free survival [7]. Now, in a recent study published in *PLOS Medicine* [8]*,* Lentini and colleagues performed a meta-analysis of 78 studies including more than 4,500 patients who received MTB-recommended treatments. The authors found that MTB-guided therapy was associated with improved clinical outcomes, including decreased risk of disease progression with risk reduction ranging from 27% to 37% depending on trial design. In addition, patients treated with MTB-recommended treatment had improved objective response rate with relative risk ranging between 1.19 and 3.32 and improved disease control rate with relative risk between 1.20 and 1.65. A progression-free survival ratio of at least 1.3 was found in 33%–43% of patients treated with MTB-recommended therapies. However, the impact on overall survival was less consistent; retrospective studies showed a significant benefit, whereas randomized controlled trials demonstrated only a non-significant trend toward improvement in overall survival.

There are limitations to this meta-analysis, including heterogeneity in the design of the included studies, the large number of retrospective studies included, selection bias, and referral bias. Finally, it is difficult to distinguish the benefit derived from precision-targeted therapies themselves and the independent contribution of the MTB process. Current evidence supports the clinical utility of MTB-guided care; however, definitive evidence demonstrating a causal benefit specifically attributable to MTB review remains limited.

In parallel with these limitations, implementing MTBs at scale brings important practical and financial challenges into focus. MTBs require significant time from highly trained professionals. This burden is particularly acute in community settings, where most cancer care is delivered. Regional or networked MTBs represent one approach to share expertise and reduce duplication of effort and have successfully collaborated with community medical oncology practices [5].

Current healthcare reimbursement structures in the US seldom compensate MTB activities, leaving institutions to absorb costs. While European adoption of MTBs varies, some countries, including Germany [9], Italy, and France now require MTB evaluation for reimbursement for targeted and off-label therapies. Given the improvements in patient outcomes afforded by MTBs, US health systems and payers could consider incentivizing MTB participation through reimbursement models. Moreover, recommendations for MTB review could be incorporated into clinical practice guidelines and MTB review could be considered by the Committee on Cancer as a quality indicator. Some experts have also called for a new oncology subspecialty focused on interpretation of molecular reports, which could be included in the MTB structure [10].

In addition to resources limitations, genomic information and genomic-directed therapies are growing exponentially, making it a challenge to keep current on recent literature. A number of artificial intelligence (AI) and machine learning tools have been developed to overcome this challenge. For example, Molecular Oncology Alamanac, which is a clinical interpretation algorithm paired with a knowledge database, was assessed in a prospective clinical trial and was able to suggest a median of two therapies per patient and identified therapeutic strategies received by 47% of patients [11]. While AI will not replace human expertise, it can augment MTB workflows by synthesizing evolving evidence and prioritizing therapeutic options. However, implementation requires careful consideration of key challenges, including: algorithmic bias, particularly if training datasets lack diversity; the need for transparency and interpretability to support clinical trust; rigorous validation in real-world settings; and appropriate regulatory oversight. Addressing these issues will be essential to safely integrate AI into MTB practice and realize its potential to enhance care delivery.

In conclusion, MTBs represent a pivotal link between the explosion of cancer genomic data and real-world patient care. The new meta-analytic evidence supports that this model of multidisciplinary precision oncology can improve patient outcomes. To fully realize the potential of MTBs, the oncology community must work to overcome implementation barriers—e.g., by securing sustainable reimbursement models, expanding the specialized workforce, and innovating to streamline workflows. Importantly, standardizing outcome measures and conducting additional large-scale trials are also essential to clearly demonstrate and further enhance the value of MTBs. MTBs can be scaled as a core component of cancer care, helping to ensure that advances in tumor genomics translate equitably into better outcomes for patients across all settings.

## Abbreviations

AI: artificial intelligence
MTBs: Molecular tumor boards
NGS: next-generation sequencing
